# Smart Sensors and Devices in Artificial Intelligence

**DOI:** 10.3390/s20205945

**Published:** 2020-10-21

**Authors:** Dan Zhang, Bin Wei

**Affiliations:** 1 Department of Mechanical Engineering, York University, Toronto, ON M3J 1P3, Canada; 2 Department of Computer Science, Algoma University, Sault Ste Marie, ON P6A 2G4, Canada; bin.wei@algomau.ca

As stated in the Special Issue call, “sensors are eyes or/and ears of an intelligent system, such as Unmanned Aerial Vehicle (UAV), Automated Guided Vehicle (AGV) and robots. With the development of material, signal processing and multidisciplinary interactions, more and more smart sensors are proposed and fabricated under increasing demands for homes, industry and military fields. Networks of sensors will be able to enhance the ability to obtain huge amounts of information (big data) and improve precision, which also mirrors the developmental tendency of modern sensors. Moreover, artificial intelligence is a novel impetus for sensors and networks, which gets sensors to learn and think and feed more efficient results back.”

The current development of sensors is purely based on mathematical models, in which one needs to know the parameters exactly. However, sometimes there are uncertainties and variation which are impossible to anticipate, and under this situation, the sensors will not work properly. By combining sensors with artificial intelligence, the uncertainties and variation can be addressed and handled. The essential thing about artificial intelligence is to predict the “future”. For example, among the applications in artificial intelligence is the learning control currently under developing. By using a learning control, uncertainties and variations can be effectively handled [1], whereas these cannot be handled traditional control systems. From another perspective, driving a vehicle at night can be used as an example. Supposing the vehicle light is not working, one can imagine that it will be difficult to drive fast as the driver’s eyes may not detect the road condition ahead. However, supposing that one drives in the daytime, it is very easy to drive fast because the driver can predict what it is going on in front of them. Our human eyes are sensors, and without the sensors, the associated systems will not work properly. To go one step further, supposing there is a GPS installed in the car, and the GPS here is considered to be artificial intelligence, by resorting to the GPS, it can help drivers drive fast even without the vehicle light at night. It is known that by integrating artificial intelligence into sensors and devices, uncertainties can be adaptively addressed so as to, for example, reduce tracking errors or improve accuracy in robotics applications, or predict the future as discussed in the previous driving case. 

This Special Issue focuses on the smart sensors and networks, especially sensing technologies utilizing artificial intelligence. This editorial summarizes the whole Special Issue. We received 43 papers in total, and 17 of them were published.

## Summary of the Special Issue

In [2], a new algorithmic rule for the purpose of streaming data, referred to as “time-aware density-based incremental local outlier detection”, was proposed to conquer variations in data that change as time goes on. The results show that the proposed “time-aware density-based incremental local outlier detection” performs better than that of the existing candidates in the sense of the AUC in most of the cases on different kinds of datasets. In [3], the study was focused on the vehicle classification with (fiber Bragg grating) FBG sensor arrays by employing AI from partial records. The developed neural network was trained by resorting to a dataset which lacked vehicle velocity data, which is generated by the visual identification of a vehicle going over the testing platform. The result indicates that the developed neural network can successfully separate trucks from other vehicles. The study shows that by using the artificial intelligence, the system can handle uncertainties even in a situation of unknown data by predicting the past events. Similarly, in [4], the study introduced a type of adaptive control algorithm for the landing gear mechanism under an unknown condition. Based on the information from the optical flow sensor and depth camera, the control system accomplished multi-sensor data fusion for the purpose of expanding the functionalities of landing and taking off for UAVs in an unknown condition and environment. 

In [5], a new unidimensional auxiliary classifier generative adversarial network was developed in order to get more signals for short-wave radio stations and the unidimensional DenseNet is employed to perceive link establishment behaviors for electronic countermeasures even with no communication protocol standard. In [6], for the purpose of fruit ripeness monitoring in real time and maximizing the fruit quality during storage via the forecasting of the current fruits condition and therefore minimizing financial loss, the study illustrated a neural network architectural design, i.e., “Xbee-based wireless sensor nodes network”, and subsequently the resulting data were used for training in the “artificial neural network” for validating the data. The results indicated that the proposed wireless sensor nodes network architecture was able to recognize the fruit condition.

In [7], the authors developed a traffic forecasting methodology which used air pollution and atmospheric parameters and the timestamped traffic intensity data for the purpose of predicting the flow of the traffic. The “long short-term memory recurrent neural network” was employed here to help predict the flow of the traffic, with the help of the data from the air pollution and atmospheric condition. Similarly in [8,9], the performance of several data processing algorithms that is geared to roadside light detection and ranging under unknown weather conditions was evaluated, and a background filtering and object clustering method was developed for the purpose of processing the roadside light detection and ranging data under the unknown weather conditions. It was shown that the current processing algorithm for the roadside light detection and ranging was based on assuming known weather conditions. Unknown weather conditions are the major challenges for data processing. 

In [10], a one-dimensional clamping force sensing approach which does not need internal force sensors was proposed for a cable-driven surgical robot. The clamping force estimation approach was developed on the basis of a “joint torque disturbance observer”, which basically examines the differences among the real-time estimated cable tension and the actual cable tension by resorting to a “PSO Back Propagation Neural Network”. The advantage of the proposed approach was that it only uses the known data of the motor displacement and without the information from the internal force sensors. In [11], a fuzzy functional dependency was used under the situation of data fusion from an automatic identification system and over-the-horizon radars sources. The fuzzy logic approach proposed in this study was proven to be a favorable tool in handling uncertainties from different sensors. 

In [12], an audification and visualization system used for people who are deaf and blind in autonomous vehicles was developed by using deep learning. The audification and visualization system has three different sectors. The data collection and management sector keeps the vehicle data, the audification conversion sector contains a speech-to-text sub-sector which can accept user speech and subsequently transform it into text data, and the data visualization sector can conjure up the collected data and set the envisaged data according to the vehicle display size. Similarly, in [13], a scenario-generation approach contingent on deep learning was developed for the purpose of automatically generating scenarios to train autonomous vehicle smart sensors. To create different situations, the developed approach extracts several events from a video that were captured on a real road based on deep learning and creates different scenarios in a virtual simulator. The method developed here contains different scenarios by extracting them from one driving event and allows interactions between objects. 

In [14], the authors developed two distributed task assignments for wireless sensor networks on the basis of the “transmission-oriented reliable and energy-efficient task allocation” for the purpose of solving the distributed task allocation issue in wireless sensor networks. In the first distributed task assignments for wireless sensor networks, the sink allocates reliability to all cluster heads based on the requirements of the reliability, in this way the cluster head carries out the local task allocation. Similarly, the global view was achieved through collecting local views from multiple sink nodes. In [15], a tracker derived from the “structural patch response fusion under correlation filter and color histogram” was developed. The developed approach contains different sub-trackers that can adaptively address illumination variation. To recognize and fully use the patches, an adaptive hedge algorithm was developed for the purpose of hedging the patches responses into a much more reliable one in each component tracker. 

In [16], a mechanical sensor-controller-integrated system was developed for the purpose of reaching the objective of identifying gait. The system contained a sensing section and a mechanical executing section. The sensing section contained a sensor which can possess two input channels. Because the system was linked with the spring, the sensor was evaluated along with the controller. 

In [17], the authors developed a convolutional neural network combined with long short-term memory networks algorithm, which had the merits of both. The developed algorithm initiated a shallow convolutional neural network to obtain the main characteristics of the molten pool image. After that, the main characteristics were converted into the feature matrix. The good aspect about the algorithm lies in the fact that it is able to learn the best hybrid characteristics via the “error back propagation algorithm” in the shallow convolutional neural networks for a single molten pool image in order to have the engineering requirements for the purpose of observing the welding process in real time. Very similarly in [18], an AI-based approach was developed for the purpose of reducing the noise in the MEMS inertial measurement unit output signals. Particularly, a derivative of the “recurrent neural network long short-term memory” was used in order to filter the MEMS gyroscope outputs, the signals of which are considered as time series.

All of the above studies indicate the significance of combining AI with sensors to address the uncertainties and unknown data. This editorial is to summarize the whole Special Issue and act as a formal closure.
